# Supplementary material for: Sex-specific variation in the use of vertical habitat by a resident Antarctic top predator
Source: Proc Biol Sci. 2020 Oct 21;287(1937):20201447. doi: 10.1098/rspb.2020.1447 (PMC7661299; doi:10.1098/rspb.2020.1447)
Supplement: Supplementary Material S1 [file rspb20201447supp1.pdf]

Supplementary Material S1: Descriptive statistics, individual maps, and data collection rules

Theoni Photopoulou, Karine Heerah, Jennifer Pohle and Lars Boehme

The material presented here is a supplement to “Sex-specific variation in the use of vertical habitat by a resident Antarctic top predator” by Theoni Photopoulou, Karine Heerah, Jennifer Pohle and Lars Boehme (2020) Proceedings of the Royal Society B (http://dx.doi.org/10.1098/rspb.2020.1447).

Seal ID	Sex	Start date	End date	Dive duration (min)	Maximum dive depth (m)	Hunting depth (m)	Proportion of dive time spent hunting	$\frac{Huntingdepth}{Bathymetry}$	Dives without hunting
ct70-356-11	M	08 Feb	22 Feb	15.5±7.1 (0.5-27.0)	331±178 (5-574)	295±186 (5-574)	0.46±0.17 (0.00-0.81)	0.57±0.36 (0-1.00)	4/168
ct70-486-11	M	09 Feb	03 Aug	6.6±6.9 (0.5-29.0)	112±158 (5-594)	99±148 (5-569)	0.51±0.19 (0.00-0.94)	0.23±0.36 (0-1.00)	18/1038
ct70-488-11	F	10 Feb	07 Oct	12.5±9.3 (0.5-50.3)	187±190 (5-844)	171±187 (5-724)	0.54±0.18 (0.00-0.97)	0.39±0.42 (0-1.00)	14/1700
ct70-490-11	M	09 Feb	01 Oct	6.1±7.0 (0.5-28.5)	101±160 (5-694)	93±157 (5-689)	0.50±0.20 (0.00-0.94)	0.19±0.35 (0-1.00)	15/839
ct70-491-11	M	15 Feb	13 Oct	11.1±8.3 (0.5-47.3)	161±219 (5-874)	128±203 (5-809)	0.44±0.24 (0.00-0.94)	0.25±0.35 (0-1.00)	59/1683
ct70-499-11	M	11 Feb	10 Nov	7.7±7.4 (0.5-37.3)	140±170 (5-724)	122±160 (5-639)	0.46±0.19 (0.00-0.94)	0.29±0.38 (0-1.00)	35/1367
ct70-500-11	M	11 Feb	03 Oct	8.1±7.2 (0.5-48.3)	162±175 (5-574)	149±169 (5-549)	0.46±0.18 (0.00-0.94)	0.35±0.40 (0-1.00)	24/1033
ct70-501-11	F	08 Feb	10 Oct	9.2±6.7 (0.5-39.3)	121±119 (5-584)	101±107 (5-584)	0.55±0.19 (0.00-0.97)	0.04±0.07 (0-1.00)	24/1555
ct70-502-11	F	09 Feb	27 Mar	9.0±7.1 (0.5-40.3)	192±183 (5-594)	177±178 (5-594)	0.45±0.18 (0.00-0.97)	0.41±0.42 (0-1.00)	14/506
ct70-503-11	F	08 Feb	19 Sep	7.7±5.3 (0.5-34.3)	129±115 (5-614)	110±104 (5-614)	0.45±0.21 (0.00-0.90)	0.09±0.16 (0-1.00)	49/1175
ct70-526-11	M	10 Feb	01 Jun	8.7±7.7 (0.5-34.3)	152±165 (5-564)	140±164 (5-519)	0.48±0.19 (0.00-0.97)	0.35±0.41 (0-1.00)	26/1211
ct70-633-11	M	14 Feb	19 Sep	8.3±6.7 (0.5-28.0)	149±170 (5-654)	135±166 (5-654)	0.45±0.21 (0.00-0.87)	0.25±0.33 (0-1.00)	34/871
ct70-634-11	M	10 Feb	12 Jul	10.6±8.7 (0.5-45.3)	188±182 (5-1269)	173±178 (5-569)	0.50±0.17 (0.00-0.90)	0.42±0.43 (0-1.00)	24/1364
ct70-637-11	F	14 Feb	08 Oct	7.8±5.7 (0.5-34.3)	119±133 (5-844)	98±119 (5-519)	0.49±0.21 (0.00-0.94)	0.10±0.15 (0-0.98)	24/879
ct70-638-11	F	09 Feb	24 Aug	5.2±4.5 (0.5-23.5)	83±109 (5-461)	72±101 (5-421)	0.50±0.22 (0.00-0.94)	0.10±0.23 (0-1.00)	22/1018
ct70-640-11	F	13 Feb	29 Oct	9.4±9.2 (0.5-95.3)	122±153 (5-614)	102±140 (5-609)	0.51±0.21 (0.00-0.97)	0.20±0.30 (0-1.00)	28/1356
ct70-642-11	F	11 Feb	04 May	7.0±5.4 (0.5-25.0)	147±162 (5-704)	127±156 (5-699)	0.42±0.22 (0.00-0.93)	0.26±0.33 (0-1.00)	46/910
ct70-643-11	F	12 Feb	01 Nov	7.1±5.8 (0.5-28.5)	85±106 (5-594)	70±96 (5-564)	0.52±0.20 (0.00-0.90)	0.03±0.08 (0-1.00)	26/1308
ct70-650-11	F	10 Feb	28 Jul	10.8±8.7 (0.5-38.3)	184±194 (5-734)	165±188 (5-729)	0.50±0.19 (0.00-0.97)	0.35±0.40 (0-1.00)	22/1190

Table S1.1. Descriptive statistics regarding dive parameters from the individual Weddell seals used in the main analysis. All seals were instrumented in the southern Weddell Sea and all dates refer to the year 2011. Dive parameters are presented as mean ± 1 standard deviation and the range is given in brackets in grey. Some dives did not have any segments that were identified as hunting segments using the methods developed by [1], these number of these dives is shown in the last column as a fraction of the total number of dives recorded for each animal. When no hunting segments were recorded there are missing values for hunting depth, proportion of dive spent hunitng, environmental variables (temperature and salinity) and the proportion of bathymetry reached.

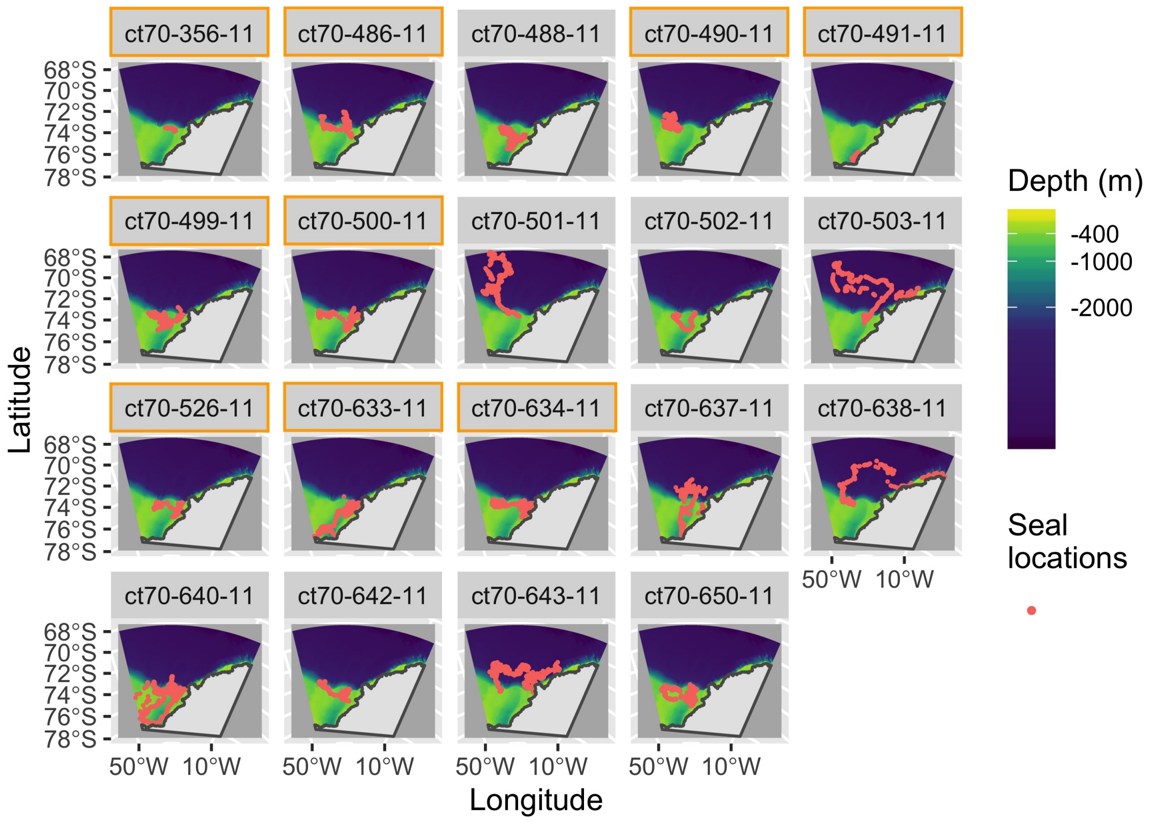

Figure S1.1. Individual satellite tracks from 19 instrumented Weddell seals carrying CTD-SRDL tags deployed in February 2011. The subplot labels are colour-coded by sex: the plots with a gold border around the heading come from male seals, the rest from females. Dive characteristics are detailed in Table S1. The background colour represents the bathymetry (depth in metres at a 0.5km resolution). The colour bar has been scaled so that yellow areas represent the continental shelf and dark blue areas represent the deep ocean. These are predicted locations from a correlated random walk model fitted to the original data, accounting for the estimated location error provided by CLS Argos, using the `foieGras` package in `R` [2].

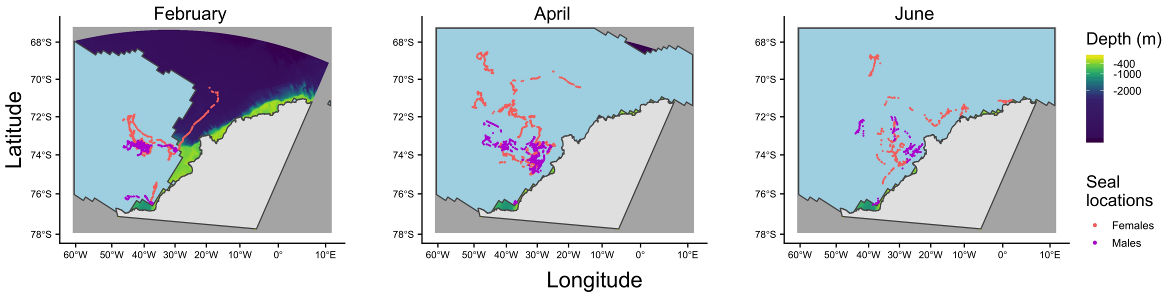

Figure S1.2. Satellite tracks from the instrumented Weddell seals shown in Figure S1 above, overlayed with sea ice extent. We show snapshots of monthly sea ice and tracks (Februrary, April and June), corresponding to the representation of the results in Figure 3 of the main text: late summer, autumn and midwinter. Sea ice information consisted of monthly sea ice extent data, accessed from the National Snow and Ice Data Centre [3]

S1.1 Data collection rules

The beginning of a haulout record is triggered when the tag is dry for at least 10min - the animal is considered to be hauled out on ice or land. A surface event is triggered when the tag is wet but there has been no dive for at least 9min - the animal is considered to be in the water but not diving). A dive event is triggered when the tag is wet and the depth is greater than 6m for at least 8sec - the animal is considered to be actively diving. An detailed explanation of how behavioural records are collected by CTD-SRDLs is provided in [4].

References

[1] Heerah K, Cox SL, Blevin P, Charrassin JB. Validation of Dive Foraging Indices Using Archived and Transmitted Acceleration Data: The Case of the Weddell Seal. Frontiers in Ecology and Evolution. 2019;7(30):1–15.

[2] Jonsen I, Patterson T. foieGras: Fit Continuous-Time State-Space and Latent Variable Models for Filtering Argos Satellite (and Other) Telemetry Data and Estimating Movement Behaviour. CRAN; 2019. R package version 0.4.01. Available from: `https://cran.r-project.org/package=foieGras`.

[3] Fetterer F, Knowles K, Meier WN, Savoie M, Windnagel AK. Sea Ice Index, Version 3. [G02135, Sea Ice Extent polygons used, accessed 08/10/2020]. NSIDC: National Snow and Ice Data Center, Boulder, Colorado USA; 2017.

[4] Photopoulou T, Fedak MA, Matthiopoulos J, McConnell B, Lovell P. The generalized data management and collection protocol for Conductivity-Temperature-Depth Satellite Relay Data Loggers. Animal Biotelemetry. 2015;3(1):21.
