# Supplementary material for: Sex-specific variation in the use of vertical habitat by a resident Antarctic top predator
Source: Proc Biol Sci. 2020 Oct 21;287(1937):20201447. doi: 10.1098/rspb.2020.1447 (PMC7661299; doi:10.1098/rspb.2020.1447)
Supplement: Supplementary Material S4 [file rspb20201447supp4.pdf]

Supplementary Material S4: Supplementary methods

Theoni Photopoulou, Karine Heerah, Jennifer Pohle and Lars Boehme

The material presented here is a supplement to "Sex-specific variation in the use of vertical habitat by a resident Antarctic top predator" by Theoni Photopoulou, Karine Heerah, Jennifer Pohle and Lars Boehme (2020) Proceedings of the Royal Society B (http://dx.doi.org/10.1098/rspb.2020.1447).

S4.1 Model structure and implementation

A partially unobserved Markov chain is assumed to determine the behavioural states and the parameters of the state-dependent distributions associated with the observed variables. While the type of event (haulout, surface and dive) is known from the data, dives are not further distinguishable into types or classes from the data alone. In this HMM analysis, surface and haulout states are therefore treated as known, while the dive states are treated as unknown states and our research question relates to inferring information about the different types of dives carried out by Weddell seals. We fit the model to all seals of each sex jointly and therefore estimate one set of parameters for females and one for males. We explored fitting a single model to all seals but this model structure did not allow us to detect differences in diving behaviour and compare the effects of the covariates in females and males. By the time one has made all parameters sex-dependent it becomes more practical to fit separate models.

S4.1.1 Estimation

The HMM parameters are estimated using numerical maximisation of the likelihood, implemented in R [1], using the `nlm` function, with the computation of the covariate-dependent transition probability matrices and the forward algorithm coded in `C++`. The forward algorithm is an efficient way of evaluating the likelihood and is one reason for the popularity of HMMs – it makes them relatively fast to fit. It corresponds to a recursive calculation of the likelihood with computational costs only linear in the number of observed time points and renders numerical maximum likelihood estimation feasible [2].

S4.1.2 Choosing the number of states

Based on prior knowledge about Weddell seals' diving behaviour from the literature, and the dive records from the current dataset, we fitted HMMs with 3 or 4 diving states (i.e. 5- and 6-state HMMs, considering both dive and non-dive states). For the male dataset, the 5-state HMM fitted the data reasonable well, while the 6-state model suffered from numerical instabilities and was difficult to interpret. For the female dataset, however, the 6-state model was stable and better able to capture the more variable dive types carried out in shallow and deep water by female seals. Using a different number of dive states for females and males corresponds well with the fact that the female seals in our dataset tend to use deep water regions more, while male seals seem to avoid them (as illustrated in Figure 1, main manuscript). Very deep water does not allow for benthic dives, so we assume that diving behaviour might be different to coastal and continental shelf areas. Based on the above considerations, we present the results of the 5-state HMM for males, and 6-state HMM for females [3].

S4.1.3 Choosing covariates

The reason for including covariates in the model was to explore temporal effects on diving behaviour, so we only considered temporal covariates and included them so that they acted on the state transition probabilities. Available covariates were light level, time of day, and seasonality, which we specify as week of the year. We reasoned that because environmental conditions vary strongly throughout the

year in the Antarctic, and therefore daily effects may vary throughout the year, these covariates needed to be included as interactions with seasonality. We fitted a model including light level and week of the year during exploratory model fitting, but we found the temporal signal to be weaker than in the model with time of day and week of the year, even though the two models contain very similar information. On this basis, the model with time of day interacting with week of the year was chosen as the final model. Our reasons for choosing this model are that 1) time of day is much easier to interpret than light level, because it is a metric we use in our everyday lives, and 2) the temporal trend in diving behaviour was stronger and clearer in the model with time of day.

S4.1.4 Salinity as a state-dependent variable

As stated in the main text, we include the time series of salinity in the state process rather than as a covariate on the state transition probabilities. Our rationale is that we regard the hydrographic conditions encountered during a dive of a particular type, to be part of that movement behaviour, not an external factor influencing it. Weddell seals are long-lived, slowly reproducing large mammals with complex spatial memories. It seems unlikely that they respond mechanically to the abiotic conditions they encounter from one dive to the next.

We initially also included temperature as a state-dependent variable, but removed it because we found a complete overlap of the state-dependent distributions, suggesting it is not useful for identifying groupings in the data.

S4.1.5 Choosing initial values for the parameters

We chose initial values empirically, for each sex separately, by looking at the distributions of the observed data, and used twenty sets of starting values to ensure the algorithm found a global maximum.

S4.1.6 State decoding

We use the Viterbi algorithm to calculate the most likely state sequence for each individual [2]. This is called global decoding and is distinct from local decoding. The Viterbi algorithm is the standard algorithm to decode the unobserved states by calculating the most likely state sequence under a given HMM or, in other words, to obtain a state label for each observation in the time series.

S4.2 Likelihood of the HMMs

The likelihood of an HMM with N states and observation vectors $\mathbf{z_1}, ..., \mathbf{z_T}$ can be written as a matrix product:

$$L = \boldsymbol{\delta}\mathbf{P}(\mathbf{z_1}) \prod_{t=2}^{T} \boldsymbol{\Gamma_t}\mathbf{P}(\mathbf{z_t})\mathbf{1}' \tag{1}$$

where $\boldsymbol{\delta}$ is a row-vector containing the initial state distribution, $\boldsymbol{\Gamma_t}$ represents the $N \times N$ transition probability matrix at time point t and $\mathbf{1}$ is a row-vector of ones. $\mathbf{P}$ denotes a $N \times N$ diagonal matrix containing the values of the N joint state-dependent densities evaluated at the observation vector $\mathbf{z_t}$. We assume the observed variables to be contemporaneously conditionally independent, given the current state. Thus, for each state, the joint state-dependent density is the product of the univariate state-dependent densities which are associated with each one of the observed variables, respectively.

In this analysis, $\mathbf{z_t}$ corresponds to the vector: duration, hunting depth, proportion of dive time spent hunting, proportion of bathymetry, and salinity at hunting depth, observed at time t given that a dive event occurred. For haulout and surface events, $\mathbf{z_t}$ only contains the observed duration.

To investigate the influence of time of day and season on the diving behaviour, temporal covariates are incorporated into the transition probabilities using multinomial logit links:

$$\ln\left(\frac{\gamma_{ij}(t)}{\gamma_{ii}(t)}\right) = \beta_{0ij} + \beta_{1ij}\cos(2\pi \mathrm{hr}_t/24) + \beta_{2ij}\sin(2\pi \mathrm{hr}_t/24) + \beta_{3ij}\mathrm{week}_t + \beta_{4ij}\cos(2\pi \mathrm{hr}_t/24) * \mathrm{week}_t + \beta_{5ij}\sin(2\pi \mathrm{hr}_t/24) * \mathrm{week}_t \quad (2)$$

where $\gamma_{ij}(t)$ denotes the probability of switching from state i to state j at time t, and the probability of remaining in a state, $\gamma_{ii}(t)$, is used as a the reference category for each state $i = 1, \ldots, N$. Given we are not fitting any random effects, the log-likelihood of interest is the sum of log-likelihoods corresponding to the different seals within each sex.

References

[1] R Core Team. R: A Language and Environment for Statistical Computing. Vienna, Austria; 2019. Available from: `https://www.R-project.org/`.

[2] Zucchini W, MacDonald IL, Langrock R. Hidden Markov models for time series: an introduction using R. Chapman and Hall/CRC; 2017.

[3] Pohle J, Langrock R, van Beest FM, Schmidt NM. Selecting the number of states in hidden Markov models: pragmatic solutions illustrated using animal movement. Journal of Agricultural, Biological and Environmental Statistics. 2017;22(3):270–293.
